# Risk factors and prognosis of postoperative delirium in nonagenarians with hip fracture

**DOI:** 10.1038/s41598-023-27829-4

**Published:** 2023-02-07

**Authors:** Shengjie Zhao, Tiansheng Sun, Jianzheng Zhang, Xiaobin Chen, Xiaowei Wang

**Affiliations:** 1 Department of Neurorehabilitation, China Rehabilitation Research Center, Capital Medical University School of Rehabilitation Medicine, No. 10, JiaoMenBei Lu, Beijing, 100068 China; 2 Department of Orthopedics, The Seventh Medical Center of China General Hospital of People’s Liberation Army, Beijing, 100700 China

**Keywords:** Psychiatric disorders, Trauma

## Abstract

Hip fractures in nonagenarians is one of the great challenges for patients of this age, the family and the larger society. The purpose of this study was to investigate the risk factors and prognosis of postoperative delirium in nonagenarians with hip fracture. 199 Eligible patients were enrolled. Confusion Assessment Method (CAM) were used to identify the delirium. Logistic regressions were used to investigate the effect of 18 pre-existing conditions on postoperative delirium. Prognosis of postoperative delirium in nonagenarians with hip fracture were also be evaluated. The results indicated the following: (1) the prevalence of postoperative delirium among nonagenarians with hip fracture was 28.1% (56 of 199); (2) coexisting disease ≥ 4 (OR = 5.355, 95% CI = 1.394–9.074, P = 0.007), longer admission to operating time (OR = 1.514, 95% CI = 1.247–1.837, P = 0.000), and general anesthesia (OR = 2.086, 95% CI = 1.804–7.968, P = 0.032) were independent risk factors for postoperative delirium in nonagenarians with hip fracture; (3) nonagenarians with postoperative delirium had a predominantly high burden of perioperative complications, long length of stay, and postoperative mortality at 30 days follow-up and 1 year follow-up than the patients without postoperative delirium. The results could enable clinicians to improve outcome after operation in nonagenarians with hip fracture.

## Introduction

Delirium, characterized by a change in mental status, loss of cognitive and perceptive functions, and alterations in the sleep cycle, is a common complication in patients with hip fracture^1^. The reported prevalence of postsurgical delirium after hip fracture ranged from 28 to 61%^2^. Postsurgical delirium is associated with greater morbidity and mortality, longer length of hospital stays, and higher rates of institutionalization, and has been suggested as significant predictor of poor prognosis in hip fracture patients^3–5^. Predisposing factors, including dementia, underlying comorbidity, and precipitating events (including major trauma and anesthesia) play an important role in the occurrence of delirium. Identifying the predisposing factors is critical for risk stratification for postsurgical delirium among the elderly hip fracture. Further, a complex intervention the preventable risk factors would enable clinicians to achieve the maximum favorable outcome for patients with hip fracture.

China has the largest population in the world and is facing population aging; as a result, the risk of hip fractures increases, in particular in nonagenarians^6^. Nonagenarians with hip fractures are different from younger hip fracture patients. In one study, the group of nonagenarians with hip fracture was with more women patients, higher prevalence of heart disease, lower prevalence of COPD and diabetes than younger patients with hip fracture^7^. A recent study reported that patients older than 90 years with hip fractures had higher mortality rates per year than younger patients^8^. Baseline functional status was poorer among the nonagenarians. The prevalence of independent for transferring from bed to chair before the fracture varied between 50 and 70% depending on the different countries and districts^7^. In addition, medical complications after hip fracture, such as delirium, had been found more frequently in nonagenarian patients with hip fracture during their hospital stay^9^. Due to the reasons above, nonagenarians with hip fracture are often excluded from clinical trials. Therefore, research on hip fractures in nonagenarians highlights the urgent need for management of this subgroup, and is of great challenges to the patients, the family and the larger society.

The purpose of this report was (i) to find the prevalence of postoperative delirium in nonagenarians with hip fracture; (ii) to find which variables can predict postoperative delirium in nonagenarians with hip fracture; (iii) to find prognosis of postoperative delirium in nonagenarian patients with hip fracture.

## Methods

### Study design and setting

We performed a retrospective analysis of demographic, clinical, and delirium data in consecutive hip fracture patients at the Department of Orthopedics, the Seventh Medical Center of People’s Liberation Army, between January 2012 and December 2020. This study was approved by the Seventh Medical Center of People’s Liberation Army Institutional Review Board, all methods were carried out in accordance with relevant guidelines and regulations. And the patients or their family gave informed consent to use their data. Data including age, gender, past medical history (hypertension, coronary heart disease, diabetes mellitus, chronic obstructive pulmonary diseases, lung infection, stroke, and renal insufficiency), post-injury factors (admission to operating time, fracture type, white blood cell count, hemoglobin, and albumin), surgery related factors (type of anesthesia, amount of blood transfusion, and surgical method), delirium (time of occurrence and duration), and length of stay were obtained by reviewing their medical records. Routine follow-up visits were scheduled at 30 days and 1 year after surgery. Mortality outcomes of inpatients using the date of death were obtained from the medical records. Mortality status and walking ability at 1 year follow-up were performed by using hospital records and/or phoning the patient’s family by a dedicated person.

### Subjects

Participants who met the following inclusion criteria were included: (1) were aged ≥ 90 years; (2) walk independently or with the aid of tools before injury; (3) intertrochanteric fracture or femoral neck fracture; (4) low energy damage (a fall from standing height or lower); (5) treated surgically for single hip fracture.

Participants were excluded if they met any of the following criteria: (1) the presence of preoperative dementia; (2) pathological fracture; (3) the American Society of Anesthesiologists (ASA) rating scale was Classes V; (4) refused follow-up after discharge or with incomplete data.

Each patient was identified by two senior orthopedists and fulfilled the criteria above.

### Assessment procedures

The following assessments were all performed at the Seventh Medical Center of People’s Liberation Army.

The most widely used instrument for identification of delirium is the Confusion Assessment Method (CAM)^10,11^. CAM scores are determined by four features: (1) acute onset and fluctuating changes in mental status, (2) inattention, (3) disorganized or incoherent thinking, and (4) an altered level of consciousness. The diagnosis of delirium by CAM requires the presence of features (1) and (2) and either feature (3) or (4). After surgery, delirium based on CAM was assessed daily, and the enrolled patients were divided into delirium group and delirium-free group. Because of difficulty in identifying true delirium and the residual effects of anesthesia, assessment of delirium on the day of surgery was excepted.

The presence of preoperative cognitive impairment/dementia was determined within 24 h of admission using the Mini-Mental State Examination (MMSE). The scores ranged from 0 to 30, with higher scores indicating higher cognitive function. Normal mental status was defined as a MMSE score of 25 points or more (maximum score is 30).

Walking ability at 1 year follow-up were categories into independent (independent community or household ambulator), dependent (patient with a minimum level of mobility) and not walking (nonambulator).

### Interventions

After preoperative examination, assessment and prepare for surgery, surgical treatment was given as soon as possible according to the type of fracture. Patients with Garden type I and II femoral neck fracture had been treated with cancellous screws. Patients with Garden type III and IV femoral neck fracture underwent hip arthoplasty. Sliding hip screw (SHS) and intramedullary nail were allocated to the treatment of stable intertrochanteric (A1, A2.1) and unstable intertrochanteric (A2.2, A2.3, A3), respectively. Different rehabilitation programs were applied to postoperative patients by specialized rehabilitation therapist according to different fracture sites and treatments. The follow-up was performed by a dedicated person over the phone who were blinded to the grouping situation at 30 day and 1 year after surgery.

### Study variables

We obtained data on a wide range of factors that might contribute to delirium in patients after hip fracture surgery. 18 variables were grouped into the following categories:(1) sociodemographic data (age and gender; n = 2 variables), (2) past medical history (hypertension, coronary heart disease, diabetes mellitus, chronic obstructive pulmonary diseases, lung infection, stroke, and renal insufficiency, and number of comorbidities; n = 8 variables), (3) post-injury factors (admission to operating time, fracture type, white blood cell count, hemoglobin, and albumin; n = 5 variables), and (4) surgery related factors ( anesthesia, surgical approach, and amount of blood transfusion; n = 3 variables).

There were three kinds of variables in this model. Continuous variables: age, admission to operating time, white blood cell count, hemoglobin, amount of blood transfusion, and albumin. Dichotomous variables: gender, fracture type, anesthesia, hypertension, coronary heart disease, diabetes mellitus, chronic obstructive pulmonary diseases, lung infection, stroke, renal insufficiency, and number of comorbidities(≥ 4 or < 4). Patients receive either regional anesthesia (spinal, epidural, or both techniques combined with no sedation) or general anesthesia (intravenous, inhalational, or combined anesthetic agents). Categorical variables: type of surgery (intramedullary nail, cancellous screws, SHS, hip arthroplasty).

### Statistical analysis

We compared patients with delirium and those without using univariate analysis. Continuous variables were expressed as means ± standard deviation (SD) or median (interquartile range, IQR) according to the distribution. The Shapiro–Wilk test was used to assess normal distribution. Differences between groups were analyzed using the independent Student’s t test for normally distributed variables, and Wilcoxon rank-sum test for non-normally distributed variables. Categorical variables were shown as frequencies (percentages), and compared by χ^2^ test. Multivariate logistic regression analyses were used to identify independent risk factors for delirium in nonagenarians after hip fracture surgery. Variables eligible for inclusion in the multivariate models included those significant at P < 0.05 in univariate analyses. All statistical analyses were performed using SPSS software (version 24.0), and a P < 0.05 was considered statistically significant.

## Results

### Subjects’ characteristics

**Figure 1:**
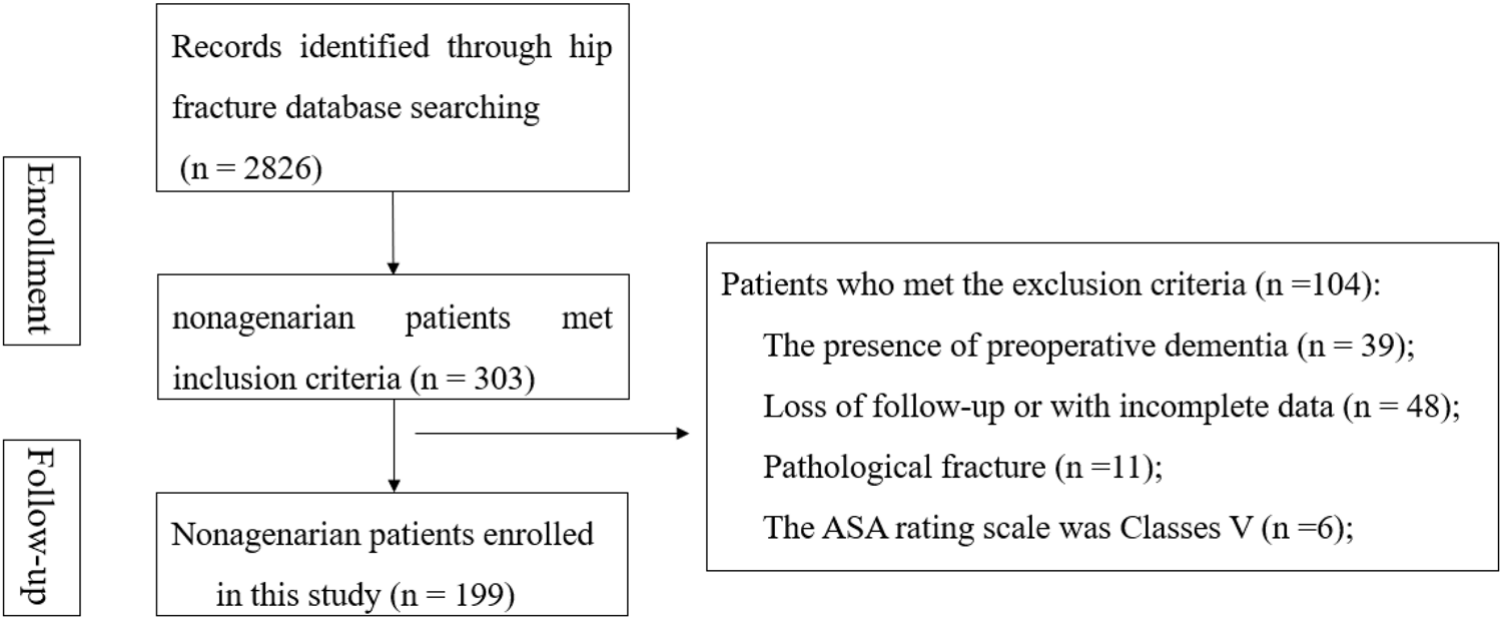
Flow diagram of the selection of patients from our hip fracture database and specific reasons for exclusion.

A flow chart of hip fracture in nonagenarians screening from the database of hip fracture in our center was presented in Fig. 1. After excluding patients who did not meet the inclusion criteria, a total of 199 patients were eligible for the study. Table 1 presents the baseline characteristics of the 199 patients included, 36.7% were men, 27.1% were general anesthesia, and 64.8% developed intertrochanteric fracture. The surgical type included intramedullary nail (122, 61.3%), cancellous screws(25, 12.6%), SHS (10, 5.0%), hip arthoplasty (42, 21.1%); The median number of subjects recruited was 92.00 years (IQR 33–482), and the median admission to operating time was 4 days (IQR3–5). In our study, the prevalence of postoperative delirium among nonagenarians with hip fracture was 28.1% (56 of 199), and was more likely to happen at 1-3d after surgery.

**Table 1:** Baseline characteristics of subjects included in the analysis.

Variables	All patients (n = 199)
Sociodemographic data	
Age (IQR), years	92 (90–94)
Gender, males, n (%)	73(36.7%)
Past medical history	
Hypertension	98 (49.2%)
Coronary heart disease	45 (22.6%)
Diabetes mellitus	24 (12.1%)
Chronic obstructive pulmonary diseases	42 (21.1%)
Lung infection	55 (27.6%)
Stroke	47 (23.6%)
Renal insufficiency	12 (6.0%)
Number of comorbidities (≥ 4)	37 (18.6%)
Post-injury factors	
Admission to operating time (IQR), days	4 (3–5)
Fracture type	
Intertrochanteric	129 (64.8%)
Femoral neck	70 (35.2%)
White blood cell count (IQR), (10^9^)	8.77 (7.09–10.29)
Hemoglobin (g/L)	104.19 ± 17.55
Albumin (IQR) (g/L)	35.75 (32.90–37.70)
Surgery related factors	
Anesthesia	
General anesthesia	54 (27.1%)
Regional anesthesia	145 (72.9%)
Amount of blood transfusion (IQR) (U)	3.50 (2.00–4.00)
Surgical method	
Intramedullary nail	122 (61.3%)
SHS	10 (5.0%)
Hip arthroplasty	42 (21.1%)
Cancellous screws	25 (12.6%)
Delirium	56 (28.1%)
Time of occurrence	
Occurs within 1–3 days after surgery	38 (67.9%)
Occurs within 4–7 days after surgery	13 (23.2%)
Occurs after 8 days	5 (8.9%)
Duration	
Symptoms last for 3 days	40 (71.4%)
Symptoms last for 4–7 days	10 (17.9%)
Symptoms last more than 14 days	6 (10.7%)

*IQR* interquartile range, *SHS* sliding hip screw.

### Comparison of nonagenarians with hip fracture in delirium group and delirium-free group

Comparing patients with and without postoperative delirium (Table 2), we found that age (P = 0.010), stroke (P = 0.032), number of comorbidities (P = 0.001), admission to operating time (P = 0.000), anesthesia type (P = 0.006), blood transfusion (P = 0.034), hemoglobin (P = 0.019), and albumin (P = 0.011) differed significantly.

**Table 2:** Factors of cohort, categorized by presence or absence of postoperative delirium.

Variables	Delirium group (n = 56)	No delirium group (n = 143)	P value
Sociodemographic data			
Age (IQR), years	92.00 (91.00–95.00)	91.00 (90.00–94.00)	0.010
Gender, n (%)			
Males	21 (37.5%)	52 (36.4%)	0.881
Female	35(62.5%)	91(63.6%)	
Walking ability before injured (%)			
Independent	26 (46.4%)	63 (44.1%)	0.762
Dependent	30 (53.6%)	89 (55.9%)	
Past medical history			
Hypertension	30 (53.6%)	68 (47.6%)	0.445
Coronary heart disease	15 (26.8%)	30 (21.0%)	0.379
Diabetes mellitus	6 (10.7%)	18 (12.6%)	0.715
COPD	15 (26.8%)	27 (18.9%)	0.219
Lung infection	17 (30.4%)	38 (26.6%)	0.591
Stroke	19 (33.9%)	28 (19.6%)	0.032
Renal insufficiency	6 (10.7%)	6 (4.2%)	0.082
Number of comorbidities (≥ 4)	19 (33.9%)	18 (12.6%)	0.001
Post-injury factors			
White blood cell count (IQR), (10^9^)	8.76 (7.02–9.84)	8.77 (7.09–10.73)	0.341
Hemoglobin (g/L)	99.49 ± 14.21	106.20 ± 18.34	0.019
Albumin (IQR) (g/L)	34.5 (4.55)	36.00 (4.42)	0.011
Fracture type			
Intertrochanteric	41 (73.2%)	88 (61.5%)	0.121
Femoral neck	15 (26.8%)	55 (38.5%)	
Admission to operating time (IQR), days	6.00 (4.00–8.00)	3.00 (3.00–5.00)	0.000
Surgery related factors			
Anesthesia			0.006
General	23 (41.1%)	31 (21.7%)	
Regional	33 (58.9%)	23 (78.3%)	
Blood transfusion (U)	4.00 (2.00–6.00)	3.00 (1.00–4.00)	0.034
Surgical approach			0.297
Intramedullary nail	40 (71.4%)	82 (57.3%)	
SHS	2 (3.6%)	8 (5.6%)	
Hip arthoplasty	8 (14.3%)	34 (23.8%)	
Cancellous screws	6 (10.7%)	19 (13.3%)	

*IQR* interquartile range, *COPD* chronic obstructive pulmonary diseases, *SHS* sliding hip screw.

### Risk factors for postoperative delirium in nonagenarians with hip fracture

All these factors were included in multivariable logistic analysis (Table 3). There was no multicollinearity (all VIFs < 10). Multivariate logistic regression identified comorbidities greater than or equal to 4 (OR = 5.355, 95%CI = 1.394–9.074, P = 0.007), longer admission to operating time (OR = 1.514, 95%CI = 1.247–1.837, P = 0.000) and general anesthesia (OR = 2.086, 95%CI = 1.804–7.968, P = 0.032) were independent risk factors of postoperative delirium in nonagenarians with hip fracture.

**Table 3:** Multivariable analysis for factors associated with postoperative delirium in nonagenarians with hip fracture.

	OR	95% CI	P-value
Age	1.063	0.935; 1.208	0.153
Stroke	1.889	0.834; 4.280	0.127
Number of comorbidities (≥ 4 vs < 4)	5.355	1.394; 9.074	0.007
Hemoglobin	0.976	0.949; 1.003	0.083
Albumin	0.948	0.858; 1.048	0.295
Admission to operating time	1.514	1.247; 1.837	0.000
General vs regional anesthesia	2.086	1.804; 7.968	0.032
Blood transfusion	1.078	0.844; 1.369	0.694

### Comparison of the outcome of patients with or without delirium in nonagenarians with hip fracture

Comparison of the characteristics of delirium group and delirium-free group in the follow-up were shown in Table 4. Nonagenarians in delirium group had a predominantly high burden of perioperative complications than in delirium-free group, as represented by 7.1% cerebrovascular events, 21.4% adverse cardiac events, and 30.4% lung infection. We found no significant differences of gastrointestinal bleeding and other complications between the two groups. The length of stay in delirium group was predominantly longer than the delirium-free group. 30 days mortality after surgery, 1 year mortality after surgery, and overall mortality were significantly higher in delirium group than in delirium-free group. May be due to similar walking ability before the fracture, which could predict activity level after 1 year, both groups had similar walking ability at 1 year follow-up.

**Table 4:** Comparison of the outcome of patients with or without delirium in nonagenarians with hip fracture.

	Delirium group (n = 56)	No delirium group (n = 143)	P-value
Perioperative complications			
Cerebrovascular events	4 (7.1%)	1 (3.6%)	0.035
Adverse cardiac events	12 (21.4%)	13 (9.1%)	0.018
Lung infection	17 (30.4%)	21 (14.7%)	0.011
Gastrointestinal bleeding	3 (5.4%)	1 (0.7%)	0.123
Others	4 (7.1%)	9 (6.3%)	0.818
Length of stay (days) (IQR)	13.00 (9.00–24.00)	11.00 (9.00–15.00)	0.024
Postoperative mortality			
30 days after surgery	8 (14.3%)	6 (4.2%)	0.028
1 year after surgery	23 (41.1%)	28 (19.6%)	0.002
Walking ability at 1 year follow-up			0.503
Independent	7/33 (21.2%)	35/115 (30.4%)	
Dependent	21/33 (63.6%)	68/115 (59.1%)	
Not walking	5/33 (15.2%)	12/115 (10.4%)	

*IQR* interquartile range.

## Discussion

Delirium is a common neuropsychiatric complication after hip fracture surgery among the elderly^12^. Based on reports in different regions, delirium is quite different among elderly hip fracture patients^13^. Reported prevalence rates of delirium among elderly hip fracture patients vary greatly due to differing definitions, tools of diagnosis, and patient populations^14^. In our study, the prevalence of postoperative delirium among nonagenarians with hip fracture was 26.1% (41 of 157), and was more likely to happen at 1-3d after surgery. Compared with elderly patients in the reported study, the incidence of postoperative delirium among nonagenarians in our study does not show a particularly high incidence^15^. The possible reasons were as follows. First, this is likely related to a selection bias where healthier nonagenarians were offered an operation. Second, a specialized department for elderly hip fractures has been established to manage this subgroup. Geriatric consultation and attendance both pre and postoperatively have been performed, which could decrease the rate of post‐operative complications. Third, patients with dementia before injury were excluded to remove the effect of dementia on delirium.

There are many risk factors for postoperative delirium^16,17^, including advanced age, dementia, anemia, dehydration, pain, anesthesia and other factors. However, this is the first study to examine risk factors associated with postoperative delirium among nonagenarians with hip fracture. Our findings suggested that coexisting disease greater than or equal to 4, having a longer admission to operating time and general anesthesia were risk factors of postoperative delirium in nonagenarians with hip fracture. Different medical comorbidities, such as dementia, hypertension, COPD, and heart failure had been found in previous studies^18–21^. The number of coexisting disease, instead of specific disease was used in our study to decrease the heterogeneity of patients. In fact, the number of comorbidities can better reflect the body's physiological reserve compared to a single comorbid disease. Under stresses such as pain, bed rest, and surgery, delirium is more likely to attack the patients with more coexisting disease, and ultimately influence the outcome. Clinically, we need to pay special attention to patients with a large number of comorbidities. The primary disease should be controlled as much as possible to reduce the incidence of postoperative delirium. Our results showed that the time interval between admission and operation was negatively associated with postoperative delirium among nonagenarians. Delay between admission and operation leads to increased long time in bed, pain, dehydration, and inverted sleep rhythm, which may result the postoperative delirium among nonagenarians. Exploring the optimal operation time for nonagenarians may reduce the incidence of postoperative delirium. At present, the relationship between anesthesia and postoperative delirium is not clear. Abbott et al^22^ showed that decrease in delirium in patients receiving regional anesthesia compared with those receiving general anesthesia. While Bryson et al.^23^ reported a relationship between postoperative delirium and certain drugs in general anesthesia, such as fentanyl and propofol. In our study, general anesthesia was one of the risk factors of postoperative delirium in nonagenarians with hip fracture. Accordingly, exposure to general anesthesia should be avoided on such patients.

Due to high mortality and fragility, nonagenarians with hip fracture completed the 1-year follow-up was rarely in the reported research. Our study confirmed that delirium was an independent risk factor of hip fracture patients at 30 days mortality after surgery, 1 year mortality after surgery, and over-all mortality, even after adjusting for various potential confounding factors. Comparing the delirium-free group, nonagenarians in delirium group had a predominantly high burden of perioperative complications, long length of stay, high overall mortality, and high mortality after surgery at 30 days follow-up and 1 year follow-up. At 1 year follow-up, we assigned similar postoperative walking ability between delirium group and delirium-free group, which had similar mobility before the fracture. The fact in elderly patients that functional status before hip fracture was important when predicting the postoperative walking ability was also applicable to nonagenarians.

There were several limitations to our study. First, our study was retrospective, prospective research should be performed to validate our findings. Second, the analysis was performed using a dataset obtained from a single center, selection bias exits. Third, the cause of death had not been analyzed.

## Conclusion

In our study, the incidence of postoperative delirium, which is more likely to happen at 1-3d after surgery and remission within 1 week, was high in patients with postoperative delirium among nonagenarians. Coexisting disease greater than or equal to 4, having a longer admission to operating time and general anesthesia were found to be statistically significant risk factors of postoperative delirium in nonagenarians with hip fracture. Comparing the control group, patients with delirium had a predominantly high burden of perioperative complications, long length of stay, high overall mortality, and high mortality after surgery at 30 days follow-up and 1 year follow-up. After adjusting for various potential confounding factors, delirium were independent risk factors of 30 days mortality after surgery and 1 year mortality after surgery for hip fracture patients (Supplementary Information).

## Supplementary Information

Supplementary Information.

## Data Availability

All data generated or analysed during this study are included in this published article and its supplementary information files.
